# A Case of Imperforated Anus

**Published:** 1793

**Authors:** William Adair

**Affiliations:** Surgeon General to the Garrison of Gibraltar.


*Gibraltar,*
*November* 3, 1792.

---

IV. *A Cafe of Imperforated Anus. By the fame.*

ON the 7th of Auguft 1792, I was called to a child of an officer of this garrifon, which had been born thirty hours without having had any evacuation by the anus. Upon examination I found a paffage of about two inches at the ufual exit of the rectum, but which beyond this was impervious. There was an appearance as if nature had made an attempt at another paffage; for at the end of the pofterior perinæum, near the os coccygis, was a fmall finus, of about half an inch, in a direction towards the rectum. In both thefe blind paffages the cuticle was equally ftrong as on any other part of the body. There could be no hefitation from which of the two we ought to attempt a comcommunication with the inteſtinal canal, and therefore, after examining with a pretty thick bougie, the former and moſt natural of theſe paſſages, I took a middling-ſized trocar, and introduced it with the point within the canula, till it reached the end; then puſhing it beyond the canula, and finding that not ſufficient, I puſhed the trocar forward, till it had completely overcome the reſiſtance. I then left the canula in, and drawing out the trocar, obſerved the point of it tinged with meconium, This latter circumſtance ſeemed to be a very favourable appearance: but after waiting ſome time nothing came away through the canula, nor when this was removed did any thing follow but a few drops of blood. I now introduced a proper-ſized bougie, which was ſuffered to remain for ſome hours; but when this was drawn out, nothing followed but a few drops of blood. A ſponge tent of a proper ſize was alſo puſhed up, and left in for ſeveral hours; but though it extended the paſſage more conſiderably than the bougie had done, no ſtools followed on its being taken out. The parts after this were left at reſt until next day, only a little warm milk was ordered to be thrown up the paſſage; and as the child (though its belly was preternaturally full, but not hard, and it had now been fifty-eight hours without any paſſage by ſtool, which in this climate, and in the month of Auguſt, was very unfavourable), ſtill ſeemed ſtrong and hearty, we introduced another pretty thick bougie up the paſſage, and left it there for twenty-four hours. This, when withdrawn was followed by a copious black ſtool, and that was ſucceeded by eight more in the courſe of twelve hours. Next day the evacuations were very frequent, to the amount of ſixteen ſtools, after which they diminiſhed in number, and became of the natural colour. The child now appeared to be going on very well, but ſoon fell off; the ſtools became ſometimes dark, and ſometimes yellow; a feveriſhneſs ſucceeded; the belly continued diſtended till two days before it died, and then became ſmaller. The child lived only fifteen days.

I had no opportunity of examining the body, ſo that the cauſe of death cannot be aſcertained; but the hiſtory of the caſe leads me to conſider it as not immediately connected with the conſequences of the operation. The child might have been defective in other reſpects eſſentially connected connected with the vital organs. The operation succeeded perfectly in giving a paſſage for the contents of the bowels; and ſo far this caſe is deſerving of notice, as it may give ſome information to others who have this operation to perform under ſimilar circumſtances. It proves that although the contents do not immediately follow the inſtrument, nor even the bougie, it is not to diſcourage the ſurgeon from future attempts, as perſevering in the uſe of the bougie may be attended by the wiſhed-for ſucceſs.

